# 175. Assessment of Institutional Uptake of Vancomycin AUC Monitoring One-Year Post Guideline Publication in Hospitals Across the United States

**DOI:** 10.1093/ofid/ofab466.377

**Published:** 2021-12-04

**Authors:** Nicole Bradley, Yuman Lee, Ariel E Francis, Duchess Iregbulem

**Affiliations:** 1 St. John’s University College of Pharmacy and Health Sciences, Queens, New York; 2 St. John’s University, Smyrna, Delaware

## Abstract

**Background:**

A new therapeutic monitoring of vancomycin for serious methicillin resistant *Staphylococcus aureus* infections guideline was published in March 2020. The guideline recommends a change in monitoring from trough to AUC/MIC based to improve patient outcomes. The purpose of this study was to determine institutional uptake of vancomycin AUC monitoring 1-year post guideline publication in hospitals across the U.S.

**Methods:**

An electronic survey was created to assess vancomycin AUC monitoring practices and distributed to the American College of Clinical Pharmacy Infections Diseases Practice and Research Network (ACCP IDprn) and American Society of Health System Pharmacists (ASHP). Initial survey distribution (phase 1) occurred May-June 2020 and aimed to serve as baseline data. The survey was re-distributed (phase 2) to the ACCP IDprn and ASHP one year later, May-June 2021. Prior to re-distribution the survey was updated to assess the impact of COVID-19 on uptake. Results were analyzed and reported using descriptive statistics. Chi-Square tests were used to compare categorical data.

**Results:**

A total of 202 responses to phase 1 and 138 responses to phase 2 were recorded. Significantly more respondents implemented AUC monitoring 1-year post guideline than at baseline (42.8% vs 29.8%, p= 0.013). In both phases, 57% of those who had not implemented AUC monitoring had plans to do so over the next year. Additionally, 46.2% phase 2 respondents reported COVID-19 impacted their ability to transition to AUC monitoring citing issues such as lack of time and inadequate resources. The most common AUC monitoring programs utilized at baseline and 1-year post guideline were purchased Bayesian software (38.3% vs. 35.6%) and homemade software (26.1% vs 23.7%). Perceived challenges to implementing AUC monitoring included cost, difficult use and integration.

**Conclusion:**

Increased uptake of vancomycin AUC monitoring occurred from baseline to 1-year post guideline publication. However, less than half of hospitals implemented this recommendation. Although COVID-19 impacted a large portion respondents’ ability to implement AUC monitoring, majority plan to transition to vancomycin AUC monitoring over the next year. AUC monitoring should be adapted by all hospitals to optimize vancomycin efficacy and safety.

**Disclosures:**

**All Authors**: No reported disclosures

